# Complex congenital cardiac anomalies in the setting of right isomerism in a 31-month-old infant: a case report

**DOI:** 10.1186/s13256-018-1835-4

**Published:** 2018-10-24

**Authors:** Frederic R. Lyimo, Pedro Pallangyo, Naizihijwa Majani, Theophylly L. Mushi, Sulende Kubhoja

**Affiliations:** 1 Department of Radiology, Muhimbili National Hospital, P.O. Box, 65000 Dar es Salaam, Tanzania; 2 Department of Pediatrics Cardiovascular Medicine, Jakaya Kikwete Cardiac Institute, P.O. Box 65141, Dar es Salaam, Tanzania

**Keywords:** Heterotaxy syndrome, Situs solitus, Right isomerism, Left isomerism

## Abstract

**Background:**

Congenital cardiac defects are not rare among neonates. Prompt assessment for life-threatening anomalies is essential for rapid management decisions and positive outcomes. Extracardiac anomalies can occur in congenital heart defects, and their presence increases morbidity and mortality in these neonates.

**Case presentation:**

We report a case of a 31- month-old infant black girl in Tanzania who presented with an on-and-off history of difficulty in breathing, easy fatigability, facial and lower-limb swelling, recurrent respiratory tract infections, and failure to thrive.

**Conclusions:**

Management of patients with heterotaxy syndrome is complex and largely depends on specific anatomy of both cardiac and noncardiac lesions. Cardiac and noncardiac management must be tailored to individual anatomy, including prophylaxis against encapsulated organisms for asplenic patients.

## Background

A birth defect is an abnormality of structure or function that originates during intrauterine life and is evident before birth or at birth or manifests later in life [1]. Congenital heart disease occurs in around 4.8–12.0 of 1000 live births in the general population. About 2.4 per 1000 cases are serious enough to require surgery or cardiac catheterization in the first year of life [2]. Prevalence of birth defects in Tanzania is estimated at 60.5 per 1000 live births; however, there is a scarcity of data for congenital birth defects owing to limited diagnostic capabilities, lack of awareness of available services, and lack of a birth defects surveillance system and registry [3].

In developed and developing countries, congenital anomalies are an important cause of neonatal mortality. There is increased mortality in patients with congenital heart disease with extracardiac malformations owing to complications associated with such anomalies, necessitating prompt diagnosis and treatment [4, 5]. The aim of this case presentation is to shed light on a rare, complex congenital heart disease and the complexity and challenges of its management in a resource-poor setting such as Tanzania.

## Case presentation

A 31-month-old infant black girl was presented to our imaging department with a recurrent history of difficulty in breathing, bluish skin discoloration, easy fatigability, failure to thrive, and on-and-off swelling of the lower limbs and face. The patient’s past medical history revealed a recurrent history of cough and fevers. She was delivered at term weighing 3.5 kg, her parents’ first-born and only child. There were no perinatal complications. The patient had an Apgar score of 9/10 and breastfed immediately. Immediately after birth, her mother started noticing that the patient was frequently inactive and weak despite breastfeeding well. The patient also started developing bluish skin discoloration a few weeks after birth, which was accompanied by easy fatigability. Milestones were delayed. She started sitting unsupported at 12 months and began crawling at 20 months. Currently, she can walk for short distances owing to the easy fatigability previously mentioned. She had several episodes of upper respiratory infections that were treated on an outpatient basis because they were not serious enough to necessitate hospital admission***.*** The patient had no family history of congenital heart disease or asthma. Her mother had no history of diabetes during the index pregnancy.

The patient’s physical examination revealed she was a girl who was small for her age, alert, and afebrile; had swollen lower limbs and puffy face; and was cyanotic with finger clubbing. Her pulse rate was 168 beats/min. Her blood pressure was 102/64 mmHg. Her respiratory examination revealed her respiratory rate was 28 breaths/min, oxygen saturation was 50%, and that she had fine bibasal crepitations. Her cardiac examination revealed S1 and S2 sounds. She demonstrated systolic murmur grade 3 at the right upper sternal border. No thrill was present. A provisional diagnosis of dextrocardia with congenital cardiac disease was made.

**Fig. 1:**
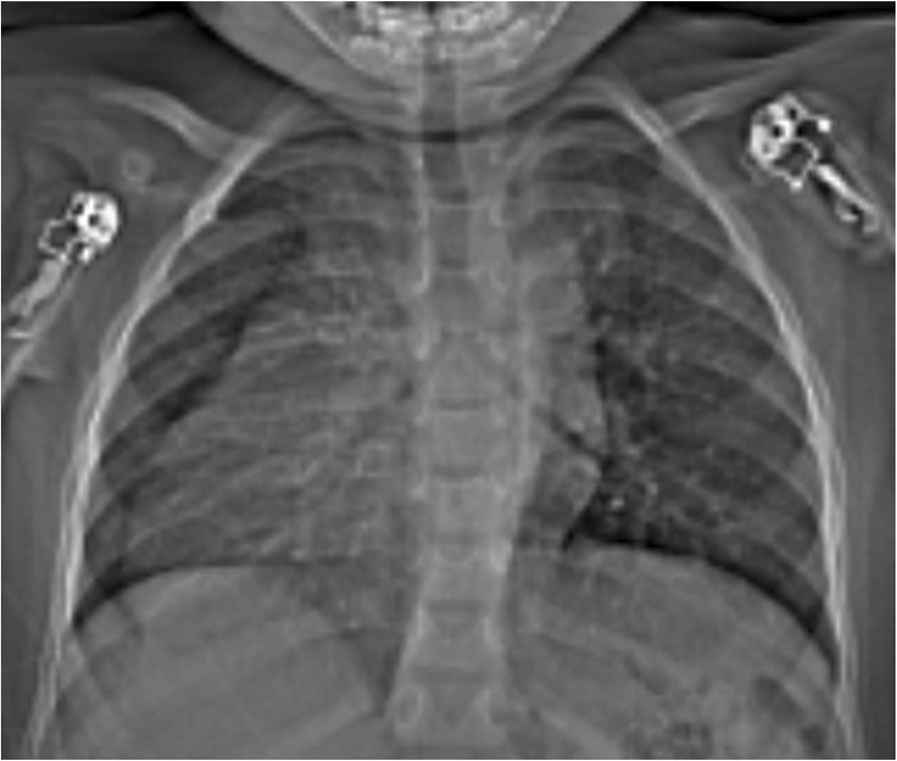
Chest scanogram showing dextroposition of the heart, widened mediastinum and trilobed lungs

Hematological tests revealed normal ranges of hemoglobin, leukocytes, and platelets. The results of the sickling test, rapid plasma reagin test for syphilis, and enzyme-linked immunosorbent assay for human immunodeficiency virus were negative. A chest x-ray revealed dextroposition of the cardiac silhouette and trilobed lungs (Fig. 1).

**Fig. 2:**
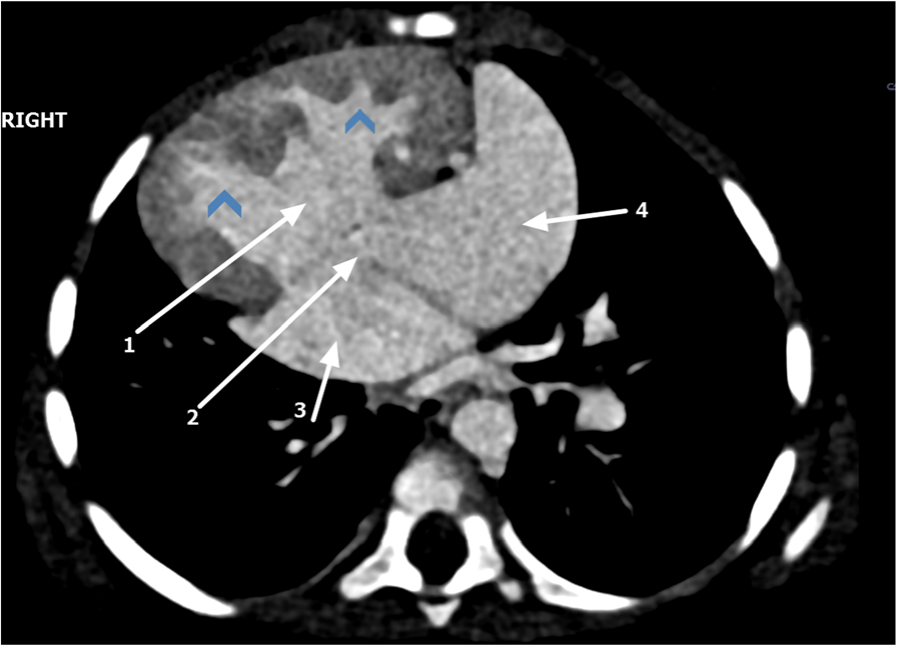
Cardiac computed tomography axial view showing dextroposition of the heart, ventricular septal defect (1), atrial septal defect (2), double right atrium (3, 4), and transposed ventricles (*arrowheads*)

**Fig. 3:**
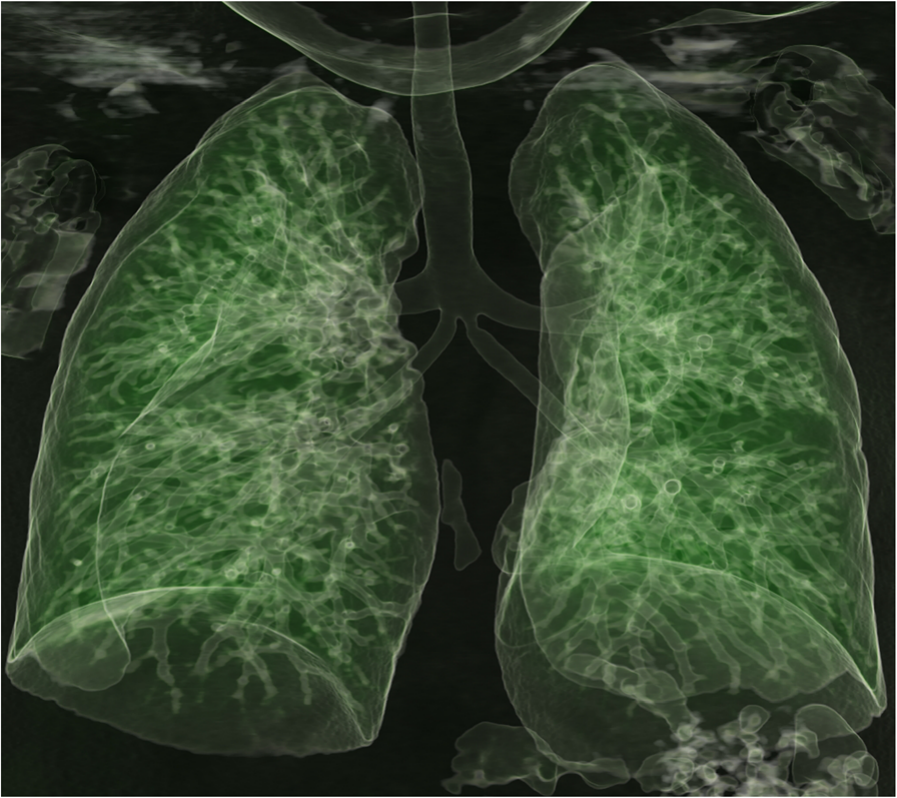
A 3D - Volume Rendering Technique (VRT) of pulmonary tree showing trilobed lungs with severely hypoplastic lower-lobe bronchi

Echocardiography revealed dextroposition of the heart, complete atrioventricular canal, pulmonary atresia, and reverse patent ductus arteriosus (PDA). The child was further investigated with cardiac computed tomography (CT), which revealed multiple complex congenital anomalies. Cardiac CT confirmed dextroposition of the heart with a huge ostium primum atrial septal defect and membranous ventricular septal defect (Fig. 2). Right atrial isomerism, bilateral trilobed lungs, and asplenia were seen. Both lower-lobe bronchi were severely hypoplastic (Fig. 3). The liver was centrally located.

**Fig. 4:**
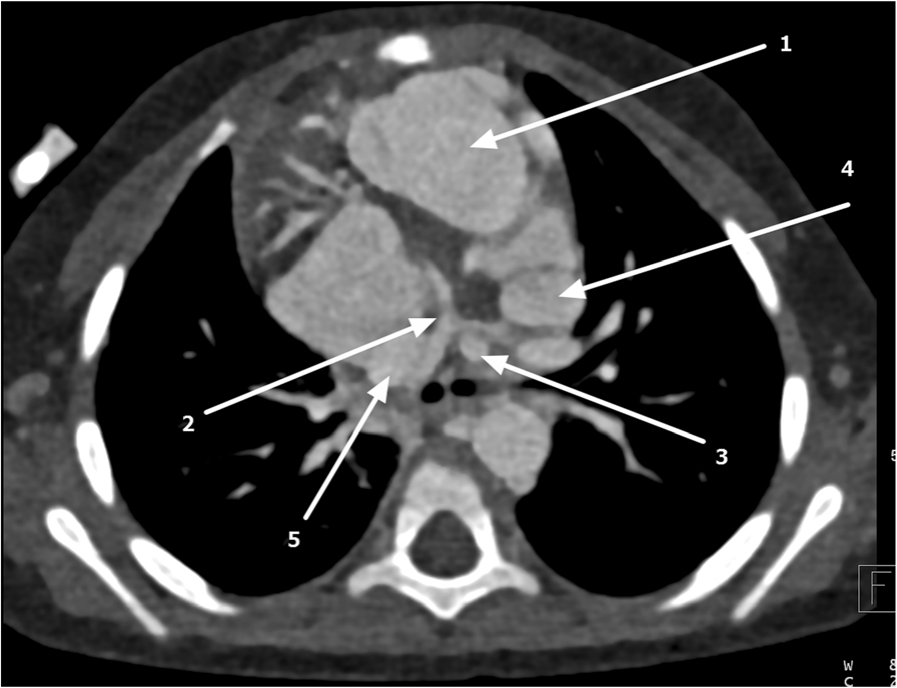
Axial cardiac computed tomography showing transposed ascending aorta 1 arising from morphologic right ventricle (1), hypoplastic main pulmonary artery (MPA) (2), left-sided patent ductus arteriosus (PDA) (3), and double-sided superior vena cava (4 and 5)

**Fig. 5:**
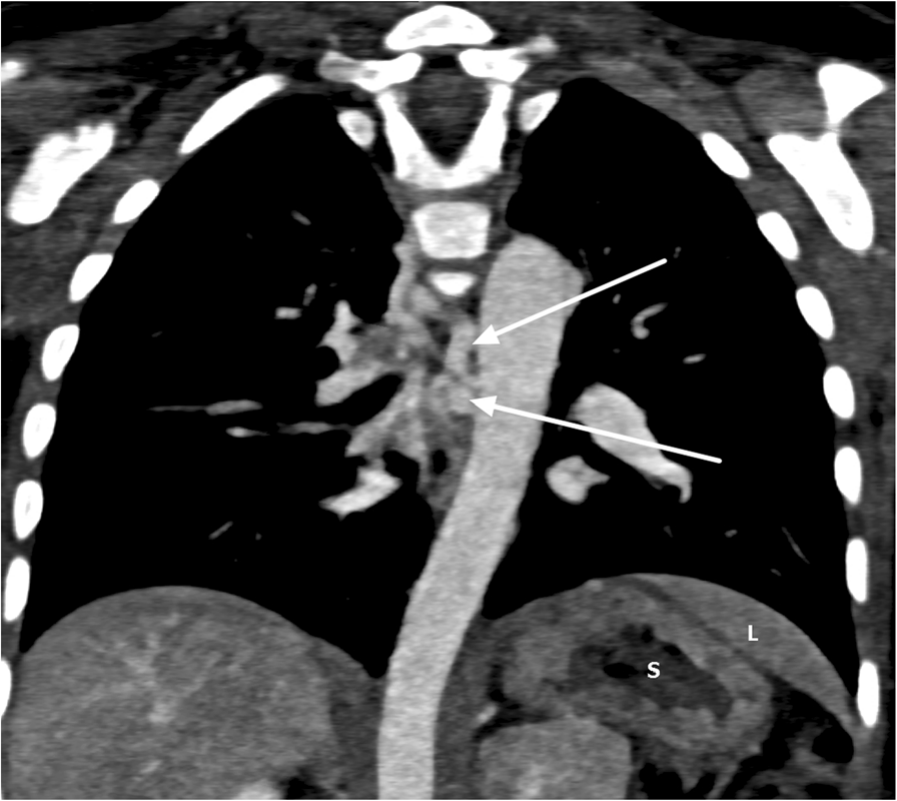
Coronal maximum intensity projection showing major aortopulmonary collateral arteries (*arrows*), stomach on the left side (S) and part of liver (L) above the stomach

Ventricular switch was seen with a morphological right ventricle on the left side giving rise to the ascending aorta. Severely hypoplastic main pulmonary artery (MPA) was seen with no direct connection to the morphologic left ventricular outlet, consistent with pulmonary atresia (Fig. 4). A left-sided PDA and multiple major aortopulmonary collateral arteries supplying the right lung were noted (Fig. 5).

**Fig. 6:**
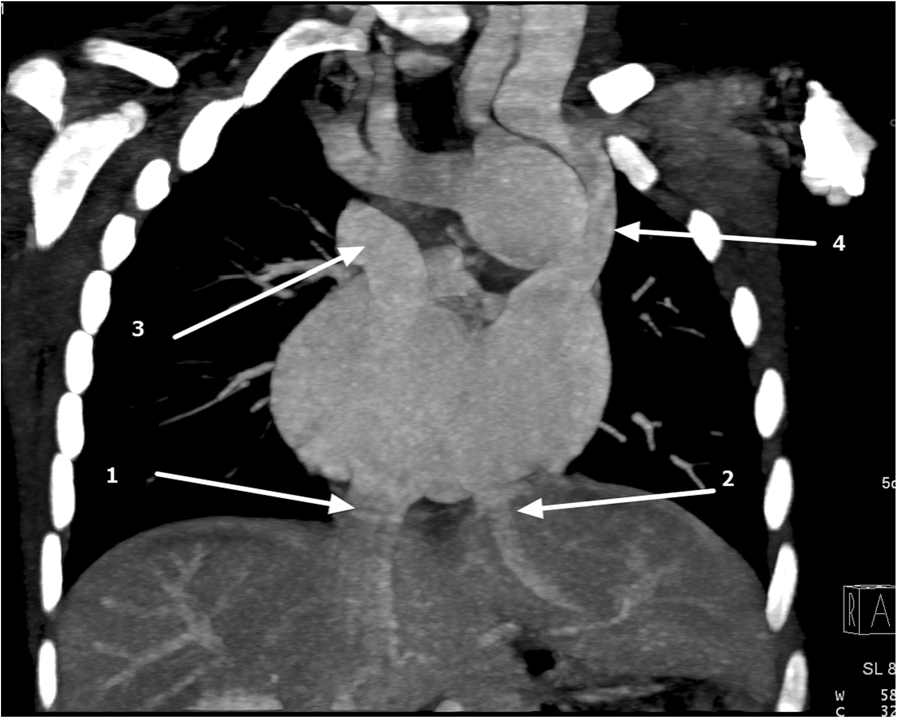
Coronal maximum intensity projection computed tomography showing asplenia, inferior vena cava (1) draining into right-sided atrium and left hepatic vein (2) draining into left-sided atrium and double-sided superior vena cava draining into separate atria (3 and 4)

Abnormal venous drainage was seen, as shown by a double-sided superior vena cava (SVC) and right upper lobe anomalous venous return. The right-sided SVC emptied into the right-sided atrium, and the left-sided SVC emptied into left-sided atrium. Liver drainage was split with the intrahepatic inferior vena cava, which received tributaries from right and middle hepatic veins, draining into right-sided atrium. The left hepatic vein drained into left sided atrium (Fig. 6).

A final imaging diagnosis of situs ambiguus with right isomerism, l-transposition of great vessels with atrioventricular discordance, ventriculoarterial discordance, dextrocardia, and multiple complex anomalies. The patient is currently on antifailure medications and prophylactic antibiotics. Owing to the complexity of the patient’s cardiac anomalies and unavailability of corrective pediatric cardiac surgical services in Tanzania, the patient was referred abroad for further management.

## Discussion

Situs solitus is the normal position of the thoracic and abdominal organs. Any disturbance in this normal arrangement of thoracoabdominal organs that does not fit with a partial or complete mirror image is termed *situs ambiguus* or *heterotaxy*. This abnormality is caused by disruption of left-right axis orientation during early embryonic development [6, 7].

Heterotaxy can be grouped into two major classes of asplenia and polysplenia syndrome or heterotaxy with right isomerism and heterotaxy with left isomerism [8]. In left isomerism or polysplenia syndrome, patients have isomeric left atrial appendages, bilaterally bilobed lungs, and multiple spleens [6, 8]. Patients with right isomerism have bilateral trilobed lungs with bilateral minor fissures and eparterial bronchi, double right atria, a centrally located liver, and a stomach in determinate position, and they lack a spleen [6, 8].

Imaging of these anomalies can be done with chest radiography, ultrasonography, CT, magnetic resonance imaging (MRI), and angiocardiography. With its direct multiplanar image capability, MRI allows detailed assessment of complex and often unexpected cardiac and extracardiac anomalies coupled with lack of ionizing radiation. CT is easily available and has very short scanning times. Precise timing needed for accurate extracardiac arterial and venous vascular imaging is possible. Technical factors can be adjusted to minimize the radiation dose to children. Essentially, the decision to image with CT versus MRI should be based on institutional equipment, scheduling, and availability as well as the patient’s ability to cooperate [9, 10].

Patients with complex cardiac lesions and heterotaxy have a poor prognosis, with mortality of over 85% for patients with asplenia and over 50% for patients with polysplenia. Infants with right isomerism frequently have associated complex cardiac congenital anomalies. Significant cardiac lesions include pulmonary atresia, common mixing situations, anomalous pulmonary venous drainage, complete atrioventricular septal defect, and ventriculoarterial discordance. Patients are immunocompromised owing to lack of a spleen and hence have an increased risk of sepsis owing to overwhelming infections in the absence of a functioning spleen [8, 11–13].

## Conclusions

Management of patients with heterotaxy syndrome is complex and largely depends on specific anatomy of both cardiac and noncardiac lesions. Cardiac and noncardiac management must be tailored to individual anatomy, including prophylaxis against encapsulated organisms for asplenic patients.

## Abbreviations

CT: Computed tomography
MRI: Magnetic resonance imaging
SVC: Superior vena cava

## References

[1] Perinatal Education Programme. Birth defects: a learning programme for professionals. Better Care: Cape Town, South Africa; 2009.

[2] Koerner A. Complex congenital heart defect, heterotaxy, imperforate anus, and other congenital anomalies in a 27-week infant: a case study. Neonatal Netw. 2014;33(4):199–203. 10.1891/0730-0832.33.4.199 24985112

[3] Kishimba RS, Mpembeni R, Mghamba JM, Goodman D, Valencia D. Birth prevalence of selected external structural birth defects at four hospitals in Dar es Salaam, Tanzania, 2011-2012. J Glob Health. 2015;5(2):020411. 10.7189/jogh.05.020411 PMC4562455 26361541

[4] Wu S, Cheng M, Zhou X, Chen Z, Huang H. GW28-e0624 Complex congenital heart disease with asplenia: a case report and review of the literatures [abstract]. J Am Coll Cardiol. 2017;70(16 Suppl):C161. 10.1016/J.JACC.2017.07.586

[5] Sarkar S, Patra C, Dasgupta MK, Nayek K, Karmakar PR. Prevalence of congenital anomalies in neonates and associated risk factors in a tertiary care hospital in eastern India. J Clin Neonatol. 2013;2(3):131–4. 10.4103/2249-4847.119998 PMC3830148 24251257

[6] Brant WE, Helms CA. Fundamentals of diagnostic radiology. 3rd ed. Philadelphia: Lippincott Williams & Wilkins; 2007.

[7] Jacobs JP, Anderson RH, Weinberg PM, Walters HL 3rd, Tchervenkov CI, Del Duca D, *et al.* The nomenclature, definition and classification of cardiac structures in the setting of heterotaxy. Cardiol Young. 2007;17(Suppl 2):1–28. 10.1017/S1047951107001138 18039396

[8] Kim SJ. Heterotaxy syndrome. Korean Circ J. 2011;41(5):227–32. 10.4070/kcj.2011.41.5.227 PMC3116098 21731561

[9] Applegate KE, Goske MJ, Pierce G, Murphy D. Situs revisited: imaging of the heterotaxy syndrome. Radiographics. 1999;19(4):837–52. 10.1148/radiographics.19.4.g99jl31837 10464794

[10] Haramati LB, Glickstein JS, Issenberg HJ, Haramati N, Crooke GA. MR Imaging and CT of vascular anomalies and connections in patients with congenital heart disease: significance in surgical planning. Radiographics. 2002;22(2):337–49. 10.1148/radiographics.22.2.g02mr09337 11896223

[11] Sadiq M, Stümper O, De Giovanni JV, Wright JG, Sethia B, Brawn WJ, Silove ED. AUDIT Management and outcome of infants and children with right atrial isomerism. Heart. 1996;75:314–9. 10.1136/hrt.75.3.314 PMC484294 8801000

[12] Agarwal H, Mittal SK, Kulkarni CD, Verma AK, Srivastava SK. Right isomerism with complex cardiac anomalies presenting with dysphagia—a case report. J Radiol Case Rep. 2011;5(4):1–9. 10.3941/jrcr.v5i4.702 PMC3303439 22470785

[13] Rubin LG, Schaffner W. Care of the asplenic patient. N Engl J Med. 2014;371:349. 10.1056/NEJMcp1314291 25054718

